# Application of Phytogenic Liquid Supplementation in Soil Microbiome Restoration in Queensland Pasture Dieback

**DOI:** 10.3390/microorganisms11030561

**Published:** 2023-02-23

**Authors:** Xipeng Ren, Maria M. Whitton, Sung J. Yu, Tieneke Trotter, Yadav S. Bajagai, Dragana Stanley

**Affiliations:** Institute for Future Farming Systems, Central Queensland University, Rockhampton, QLD 4701, Australia

**Keywords:** pasture dieback, phytogenic liquid, carvacrol, cinnamon, soil microbiota

## Abstract

Pasture production is vital in cattle farming as it provides animals with food and nutrients. Australia, as a significant global beef producer, has been experiencing pasture dieback, a syndrome of deteriorating grassland that results in the loss of grass and the expansion of weeds. Despite two decades of research and many remediation attempts, there has yet to be a breakthrough in understanding the causes or mechanisms involved. Suggested causes of this phenomenon include soil and plant microbial pathogens, insect infestation, extreme heat stress, radiation, and others. Plants produce a range of phytomolecules with antifungal, antibacterial, antiviral, growth-promoting, and immunostimulant effects to protect themselves from a range of environmental stresses. These products are currently used more in human and veterinary health than in agronomy. In this study, we applied a phytogenic product containing citric acid, carvacrol, and cinnamaldehyde, to investigate its ability to alleviate pasture dieback. The phytogenic liquid-based solution was sprayed twice, one week apart, at 5.4 L per hectare. The soil microbial community was investigated longitudinally to determine long-term effects, and pasture productivity and plant morphometric improvements were explored. The phytogenic liquid significantly improved post-drought recovery of alpha diversity and altered temporal and spatial change in the community. The phytogenic liquid reduced biomarker genera associated with poor and polluted soils and significantly promoted plant and soil beneficial bacteria associated with plant rhizosphere and a range of soil benefits. Phytogenic liquid application produced plant morphology improvements and a consistent enhancement of pasture productivity extending beyond 18 months post-application. Our data show that phytogenic products used in the livestock market as an alternative to antibiotics may also have a beneficial role in agriculture, especially in the light of climate change-related soil maintenance and remediation.

## 1. Introduction

Pasture dieback (PDB) is a rapidly spreading natural disaster in Australia. For decades, the causes of the dieback have not been identified. PDB results in a decline in pastureland, leading to a drop in the quality and quantity of grazing pasture for livestock and negatively impacting the Australian economy and the environment. According to a report by the Australian Bureau of Statistics [1], 129 out of 172 million hectares of Queensland is utilised for grazing. The dieback poses a serious threat to the productivity and sustainability of grazing producers. The Department of Agriculture and Fisheries [2] project report in 2016 reported that 35,000 hectares are affected across 120 properties in Queensland. However, PDB has worsened during the last five years, in which the affected pasture is up to 4.4 million hectares [3], spanning from Townsville in Queensland to northern New South Wales. 

The condition was first noted in buffelgrass (*Cenchrus ciliaris*) in the 1990s and is now associated with several tropical and subtropical grasses in both imported and native species. Several direct remediation trials attempting to restore affected soil by re-sowing, burning, fertilising, and slashing have not succeeded. Current knowledge suggests PDB is a multifaceted problem affecting the grass ecosystem, with soil, bacteria, fungi, helminths, insects, and other communities thrown out of balance. Among the various symptoms, leaves turning red from the tip towards the ligule are visually the most prominent [4]. The red colouration of the leaves diverges from bright to dark to bronze, with the oldest leaves turning red first and progressively affecting younger leaves. The symptoms of red leaves are more visible on the upper surface than on the lower surface. The roots of the affected grass grow poorly compared with unaffected plants [4], presenting smaller, thinner, softer, and darker roots compared with healthy grass, and they break easily. The plants that are affected by dieback weigh less, have fewer tillers, and have shorter leaves when compared with unaffected plants. Such effects of PDB could seriously affect pasture-based cattle production in Australia. 

Buffelgrass was introduced into Australia decades ago due to its robust adaptability to local climate and soil. Some varieties have been introduced for commercial pasture purposes, with three buffelgrass varieties being the most common: American, Biloela, and Gayndah [4]. Buffelgrass has been distributed across northern Australia and is found in southern areas. In central Queensland, PDB was first identified over 25 years ago; however, it is continuously spreading to other regions across Australia, having crossed the border of New South Wales in 2019.

Australia is one of the world’s most efficient producers of beef cattle. The high efficiency of Australian beef cattle production depends on several factors, including the quality of the grazing land, climate, and the breed of cattle. Australian beef cattle are primarily grass-fed, and the country’s climate and soil conditions are well-suited to growing grass. The Angus cattle breed is particularly well-suited to the Australian environment. Australia’s cattle production makes up approximately 2% of the global cattle herd [5] and exports more than 70% of its cattle [6]. In 2019, Australia was the second-largest beef exporter in the world, with 76% of its beef being exported according to an estimation by Meat & Livestock Australia [7]. As of June 2019, Australia had 24.7 million cattle, with Queensland (the state with the largest cattle industry) accounting for approximately 45.7% of that total, followed by New South Wales and Victoria [7]. From 2018 to 2019, Australia exported 1.3 million cattle valued at AUD $1.6 billion. Furthermore, beef consumption in Australia generates a significant economic income, estimated to be approximately AUD $7.8 billion in 2019 according to Meat & Livestock Australia [7]. The lack of progress in pasture dieback research and its rapid spread in the last few years is among the highest risks for the sustainability of the cattle industry in Queensland and Australia. 

Plant development is highly dependent on the associated microbial communities. The soil microbiome consists of all microorganisms living in symbiosis with the plant, including bacteria, fungi, and viruses. The microbiome plays a crucial role in plant development, providing essential nutrients, protecting the plant from pathogens or environmental stress, and influencing plant hormone signalling to improve the plant’s overall health [8]. Plants can recruit soil microbiomes to assist in controlling the infection induced by soil pathogens. Plants can choose or reject microbiota from the soil by removing leaves and branches or distributing root secretions [9,10]. On the other hand, plants often suffer from various microbial diseases. It has been speculated that pasture dieback may be caused by bacterial, fungal, and viral pathogens without definite evidence. 

Plants fight pathogens by producing a vast range of bioactive products. It was proposed that the sustainability of plant production can be improved through bioactive natural products [11]. However, despite the projected major benefits [11,12,13] of phytogen use as natural biopesticides in plant agriculture, bioactive products are often used more for human or animal health than plant health [14]. Some of the main reasons are high volatility and short environmental survival, resulting in the need for higher application rates [12] which increases the cost compared to chemical pesticides. In addition to rapid degradation, most studies on their efficacy in plants were completed in vitro and they are still less available as a plant treatment option [13]. With growing concerns regarding the role of pesticides and herbicides in global pollution and adverse effects on human health, as well as growing demand for natural or organic pest control methods, bioactive plant products are becoming more popular in agronomy, and a range of antifungal, antibacterial, antiviral, and insecticide products are now available on the market (reviewed in [11]). 

In this project, a phytogenic product was applied to treat pasture dieback. Phytogenic liquid was initially developed as an alternative livestock complementary feed and its formulation overcomes the volatility and persistence issues, making it a promising candidate for PDB remediation. Carvacrol has potent antimicrobial activity against the common pathogens of vegetable crops [12] and has a protective role in seed storage [15]. Carvacrol, a monoterpenoid phenol in many aromatic plants, including thyme and oregano, is used as a food flavouring, additive, fragrance, and preservative [16]. Carvacrol also has a range of biological actions, including anti-cancer, neuroprotective, immunopromoting, antibacterial, antifungal, antiviral, and insecticide effects [17,18,19,20,21,22]. Carvacrol shows antimicrobial activity against major plant pathogens, such as *Fusarium oxysporum* [15], *Cladosporium herbarum* [23], and *Penicillium* spp. [24,25], and was recommended as a potent antifungal agent. 

Cinnamaldehyde, an organic compound naturally occurring in the bark of cinnamon trees, gives the cinnamon spice its flavour and odour [26]. Studies indicate its anti-inflammatory, antibacterial, and immune-modulating properties [27,28,29]. It is highly regarded for its antimicrobial and range of medicinal properties (reviewed in [30]). Citric acid has a remarkable range of benefits in plant and soil health. It alleviates heavy metal toxicity by increasing plant biomass, photosynthesis, and growth, and is one of the most commonly used chemicals for phytoremediation. Citric acid also promotes stress tolerance in plants [31] that spans the range of abiotic stresses, such as acidity, drought, salinity, temperature, and heavy metal stresses (reviewed in [31]). Citric acid is beneficial for the soil rhizobia community [32], and due to its growth-promoting effects it is often referred to as a plant growth hormone [33].

The literature suggests that the components present in phytogenic liquid have the unique ability to rectify an extraordinary range of possible soil and plant health issues that likely play a role in pasture dieback onset and persistence. In this study, we present evidence of the long-term beneficial effects of phytogenic liquid in pasture dieback via modification of the soil microbial community, improved plant morphometrics, and significantly higher pasture productivity extending beyond 18 months after initial use.

## 2. Materials and Methods

This trial was conducted on an organic beef farm near Rockhampton, Queensland, Australia, with stock grazing the 1400 hectares of clean, pristine floodplains of the Fitzroy River. The owner has spent 7 years developing the farm as a case study for regenerative agriculture in the beef industry. The farm has approximately 100 hectares of pasture dieback affected area, which becomes more severe after drought. As a result, cattle do not graze in those areas, reducing the pasture carrying capacity. 

The experiment consisted of 3 randomised replicates for control (CTR) and 3 for phytogenic liquid (PHY). The core components in phytogenic liquid are cinnamaldehyde, carvacrol, and citric acid (source: Activo^®^ Liquid from EW Nutrition GmbH, Visbek, Germany). Plots were 5 × 5 m blocks separated by a 2 m buffer area between plots to prevent contamination. Phytogenic liquid solution diluted at a ratio of 0.27 mL/L was sprayed twice, one week apart, and each 25 m^2^ plot received 50 L of phytogenic liquid diluted solution. This is equal to 0.54 mL/m^2^, resulting in a final application rate of 5.4 L of phytogenic liquid per hectare during each of the 2 applications, or a total of 10.8 L/ha of phytogenic liquid used. Control plots received the same amount of water instead of phytogenic liquid dilution. 

Soil core sampling was undertaken using a T-bar at 15cm depth. One sample was taken from each plot before phytogenic liquid was applied in week 0 and weekly from week 1 to week 6, then fortnightly from week 8 until week 20. All samples were immediately placed on dry ice and stored at −80 °C in freezers for further analysis.

Two random samples were collected from each plot before phytogenic liquid was applied and again in week 20 using a 50 cm × 50 cm square quadrat to randomly throw twice per plot, then everything above the ground was collected within the quadrat. The samples were kept in paper bags and then dried in the oven at 60 °C for 72 h. After 11- and 18-months post-application, the samples were retaken to confirm long-term impacts. The dried samples were weighed and then recorded to calculate dry matter using the following formula:

$$Dry\;matter\left(\mathrm{kg}/\mathrm{ha}\right)=\frac{Dry\;weight\left(g\right)}{Quadrat\;size\left(\mathrm{ha}\right)\times 1000}$$

In addition to dry matter, plant properties were recorded at week 20 and after 11 months of application (week 48), including the length and width of the longest leaf, length and width of the youngest leaf, the number of tillers, plant height, the total number of roots, length of the longest root, root thickness, and seed heads (if applicable). The measurements were taken for grass and for the most dominant weed, wild sage.

After the 1st trial was completed and the long-term effects of phytogenic liquid were observed, the opportunity presented itself to repeat the trial on another pasture dieback site at a different property. The 2nd trial aimed only to confirm the biomass and plant morphometric improvements, and soil samples for microbial communities were not collected. This trial was performed using an identical experimental setup and sampling procedures. The second dieback site presented as a paddock lesion, fast spreading in all directions, rather than random patches of dieback as was the case at the 1st trial site. Another difference between the 1st and the 2nd trial was that the 2nd trial had vast areas of the paddock not yet affected by dieback, which allowed us to sample a healthy control that was not present in the original trial. 

Soil microbial DNA was extracted using a DNA soil kit (DNeasy PowerSoil Pro Kit, Qiagen, Hilden, Germany) and the quality was assessed by NanoDrop^TM^One^C^ (Thermo Fisher, Waltham, MA, USA). The 16S DNA library was prepared by amplifying the V3-V4 hypervariable region of the 16S rRNA gene using dual index primer pairs (forward primer Pro341F 5′-CCTACGGGNBGCASCAG-3′; reverse primer Pro805R 5′-GACTACNVGGGTATCTAATCC-3′) with an Illumina linker sequence, index, and heterogeneity spacer. Agarose gel electrophoresis was used to visualise the PCR product. The 16S amplicon library was pooled and cleaned using an AMPure XP PCR purification kit (Beckman Coulter, Sydney, NSW, Australia). The sequencing of the library was outsourced to Azenta Life Sciences (Suzhou, China) and sequenced as a 2 × 300 bp paired end reads using the Illumina MiSeq system with Illumina-recommended kits and protocols. 

All plant data, including biomass and morphometrics, were analysed for significance using the Mann–Whitney test performed in GraphPad Prizm v9. Prizm was also used to plot alpha diversity indicators originally calculated using the Phyloseq R package. Distance matrices (UniFrac and Bray-Curtis) were calculated from the rooted Newick OTU tree, which was obtained in Quantitative Insights Into Microbial Ecology 2 (QIIME 2). Ecological data interactions with the microbial community were analysed using Microeco R package. Permanova+ was used in Primer-e v7 [34] to calculate Simper and PERMANOVA data. 

## 3. Results

### 3.1. Soil Community Structure

**Figure 1:**
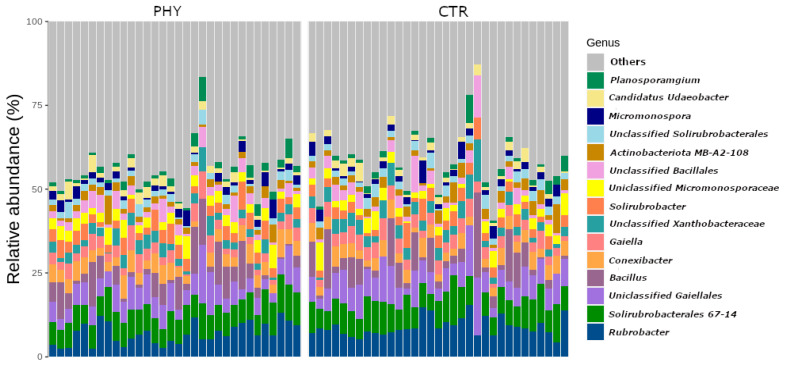
Relative abundance of top 15 genera from CTR and phytogenic liquid.

The soil bacterial community of the pasture dieback paddock had a relatively unusual pasture bacterial community. The most abundant 15 genera, based on rarefied sequence counts, were *Rubrobacter*, *Solirubrobacterales 67-14*, unclassified *Gaiellales*, *Bacillus*, *Conexibacter*, *Gaiella*, unclassified *Xanthobacteraceae*, *Solirubacter*, unclassified *Micromonospora*, unclassified *Bacillales*, *Actinobacteriota MB-A2-108*, unclassified *Solirubacterales*, *Micromonospora*, *Candidatus Udaeobacter*, *Planosporamgium* (Figure 1). Among them, *Rubrobacter* and *Solirubrobacterales 67-14* were dominant genera in both the PHY and CTR groups. *Actinobacteriota* dominated both treatment communities, with an average relative abundance of 66.7% in the PHY group and 69.2% of the total sequence counts in the CTR group, followed by *Firmicutes* and *Proteobacteria* (Figure S1). Both groups also had a range of less abundant phyla, including *Gemmatimonadota*, *Verrucomicrobiota*, *Myxococcota*, *Methylomirabilota*, *Entotheonellaeota*, *Planctomycetota*, *Nitrospirota*, and others, shown in Figure S2.

### 3.2. Alpha Diversity

**Figure 2:**
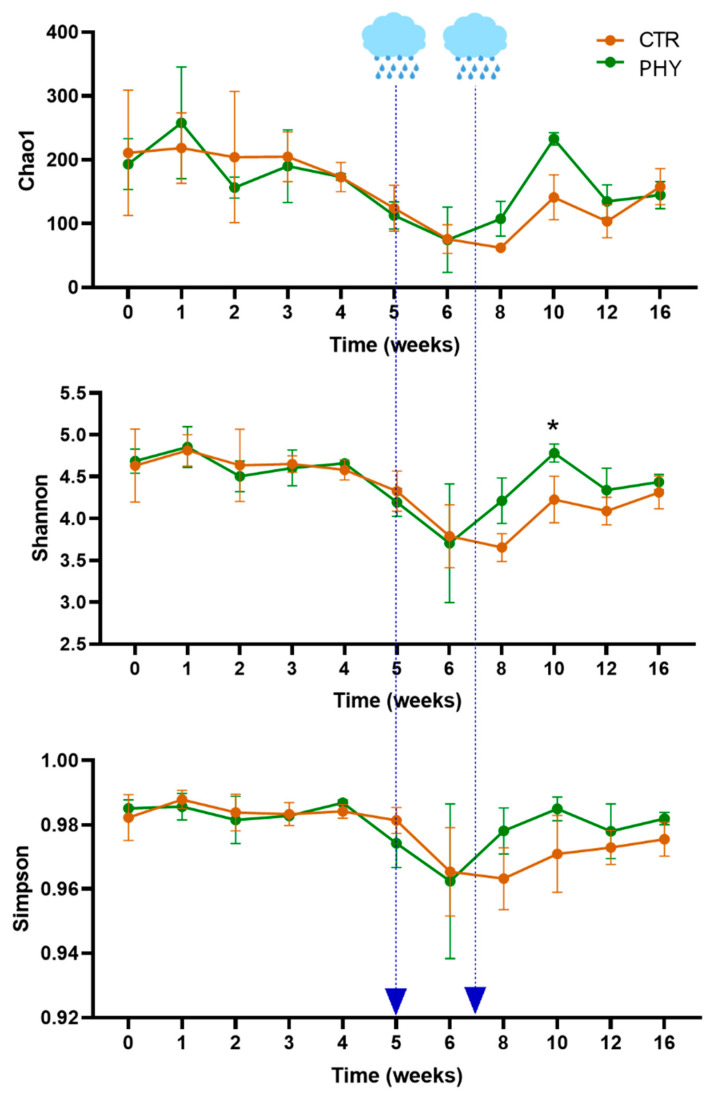
The temporal plot of alpha diversity indexes. The arrows point to the two significant rain events in weeks five and seven. The asterisk indicates a significant (*p* = 0.04) difference in the Shannon diversity index at week 10.

The PHY supplementation did not significantly affect any of the calculated alpha diversity indexes (Observed Species, Chao1, Shannon, Simpson, Fisher, Ace, Dominance, and Evenness). However, interesting temporal trends were observed, showing that while there were no changes in the diversity profile during drought weeks after the rain event, both richness and diversity were restored faster in PHY-treated plots (Figure 2). This field trial started during the drought season and only had two significant rain events during weeks five and seven. There was no more noteworthy rain between weeks 7 and 16 when the soil sampling stopped.

### 3.3. Spatial and Temporal Alterations in Beta Diversity

In order to investigate the beta diversity variable significance, we used the Primer-e v7 PERMANOVA function. We set up the PERMANOVA design to account for the longitudinal, temporal nature of the data (weeks) and nested replicate plots (spatial difference) in the treatment group and investigated the significance and interactions of the main variables: treatment (CTR vs. PHY), time (weeks), and replicate plots. We analysed the OTU-level data using weighted and unweighted UniFrac, and phylum- and genus-level data using a square root transformed abundance-based Bray–Curtis similarity matrix (Table 1).

All four PERMANOVA analyses agree with the high significance of temporal fluctuations in bacterial microbial communities; the plot was also significant at all levels except phylum. Phytogenic liquid treatment significantly affected the phylum-level taxa, but its effects on microbiota were fundamentally masked by the power of the temporal and, to a lower level, spatial differences (between replicate plots). Genus-level data showed significant interactions between treatment and time variables, indicating that genus-level alterations due to phytogenic liquid supplementations varied temporarily. This is in agreement with observed fluctuations of the most influential genera shown in Figure S3, where similar temporal patterns influenced by drought/rain as in alpha diversity (Figure 2) were observed. 

The taxa (phylum and genus level) were then investigated with the highest contribution to phytogenic liquid-driven community alterations using the SIMPER algorithm in Primer-e v7 (Table 2). The top phyla contributing to the dissimilarity between the CTR and phytogenic liquid-treated soil communities were *Firmicutes* (19.38%), *Proteobacteria* (10.14%), and *Actinobacteriota* (9.59%), cumulatively making up 39.11% of CTR–PHY group dissimilarity.

**Figure 3:**
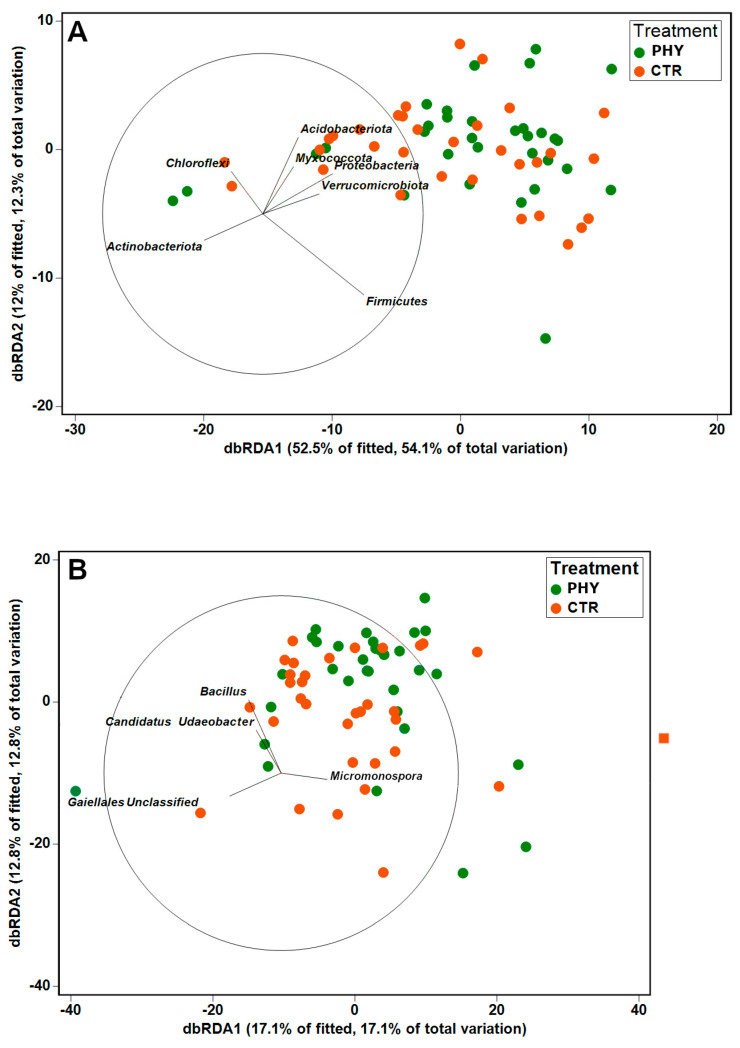
dbRDA plot at a phylum (**A**) and genus (**B**) level. Only the top taxa explaining the difference between the treatments are shown as loading vectors depicting their relative contribution in predicting the differences between PHY and CTR.

The SIMPER analysis is cross-validated using dbRDA plots (Figure 3), where the dbRDA algorithm selected the same genera as SIMPER as drivers of the CTR–PHY dissimilarity. 

### 3.4. Temporal Correlation

**Figure 4:**
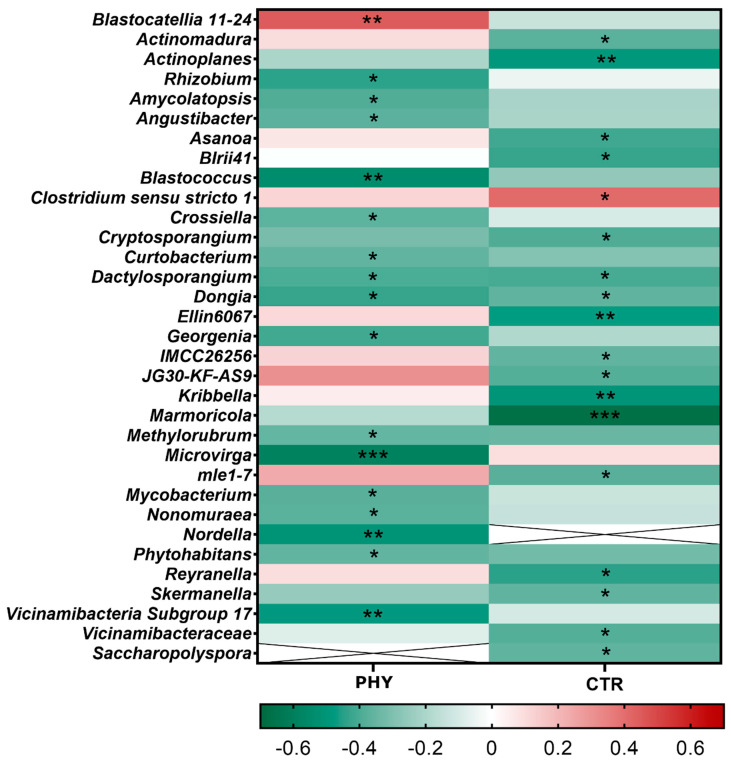
The temporal correlation heatmap. White cells with the x mark represent the genus not detected in that group. Green cell colour indicates negative temporal correlation and red cell colour indicates positive temporal correlation in each group, as shown in the heatmap correlation bar. * = *p* < 0.05; ** = *p* < 0.01; *** = *p* < 0.001.

The Microeco R package was used for the temporal correlation analysis. The analysis was performed separately in CTR and phytogenic liquid-treated groups (Figure 4). The addition of phytogenic liquid prevented the highly significant depletion of *Marmoricola* and increased the abundance of *Blstocatellia* genus *11-24*, which was slightly reduced in CTR. Phytogenic liquid also strongly suppressed *Microvirga* which was very slightly positively correlated with time in CTR. These temporal correlation differences agree with the significance of the treatment–time PERMANOVA interaction at the genus level discussed above.

### 3.5. Taxa Responding to Phytogenic Liquid Application

**Figure 5:**
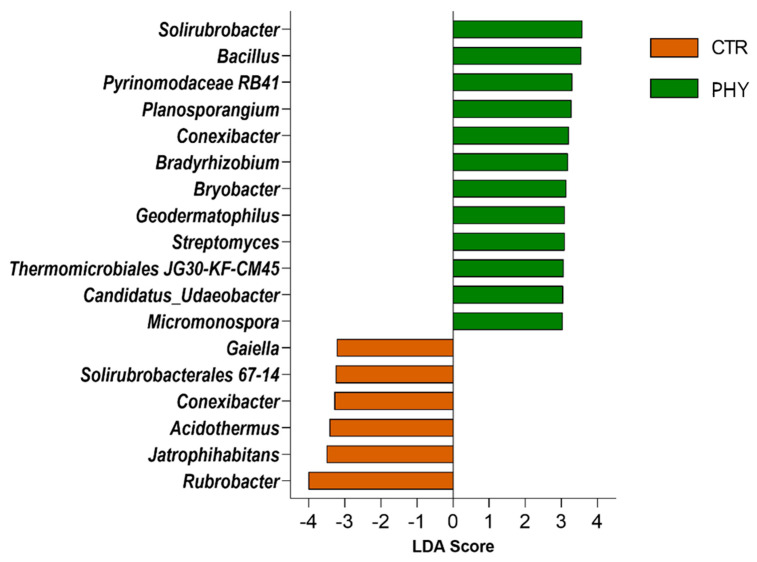
LEfSe plot at the genus level shows significantly altered (*p* < 0.05) genera with an absolute LDA score > 3.

LEfSe was used to detect taxa responding to the phytogenic liquid application at the genus level (Figure 5). *Rubrobacter*, *Jatrophihabitans*, *Acidothermus*, *Conexibacter*, and *Gaiella* were enriched in dieback, while *Solirubrobacter*, *Bacillus*, *Planosporangium*, *Conexibacter*, *Bradyrhisobium*, *Bryobacter*, *Geodermatophilus*, *Streptomyces*, *Thermomicrobiales*, *Candidatus Udaeobacter*, and *Micromonospora* were enriched in soil treated with phytogenic liquid (Figure 5).

### 3.6. Interactions with Minerals

**Figure 6:**
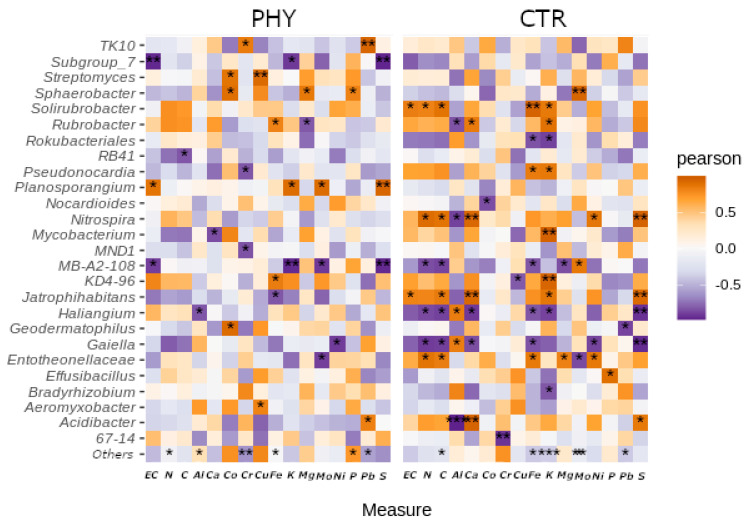
Genus abundance and soil parameter correlation heatmap. * = *p* < 0.05; ** = *p* < 0.01; *** = *p* < 0.001.

There were no significant differences in mineral concentrations between CTR and PHY plots 12 weeks post-application. On the other hand, there were significant correlations between the concentration of some minerals and taxa abundance, often opposite in CTR and PHY-treated plots. The heatmap in Figure 6 shows mineral–genus correlations demonstrating the influence of phytogenic liquid on soil taxa–mineral interactions; for example, *Solirubrobacter* and *Jatophihabitans* are significantly positively correlated with electric conductivity (EC) in CTR, while they are marginally negatively correlated with EC in the PHY group. *Mycobacterium* was significantly negatively correlated with Ca in PHY and marginally positively correlated in CTR, while *Jatophihabitans* is highly significantly correlated with Ca in CTR and negatively correlated in PHY. *Streptomyces*, *Sphaerobacter*, and *Geodermatophilus* are significantly positively correlated with Co in the PHY and negatively correlated in CTR plots. *Streptomyces* correlate significantly positively with Cu only in PHY and marginally negatively in CTR. *Sphaerobacter* is significantly positively correlated with Mg in phytogenic liquid and negatively in CTR. *Acidibacter* was significantly positively correlated with Pb in PHY while negatively correlated in CTR. *Planosporangium* was strongly and significantly positively correlated with S in the PHY and marginally negatively correlated in the CTR group. 

There are more examples of such opposite genus–mineral correlations, but *Haliangium* and *MB-A2-108* have shown double-significant opposite effects. *MB-A2-108* was significantly negatively correlated with Mo in PHY, but significantly positively correlated with Mo in CTR plots. *Haliangium* was significantly positively correlated with Al in the control group and significantly negatively correlated with Al in the PHY group. This indicates that Al strongly stimulates *Haliangium* growth only in the presence of phytogenic liquid, and a similar interpretation can be offered for other interactions. 

### 3.7. Plant Morphometrics

**Figure 7:**
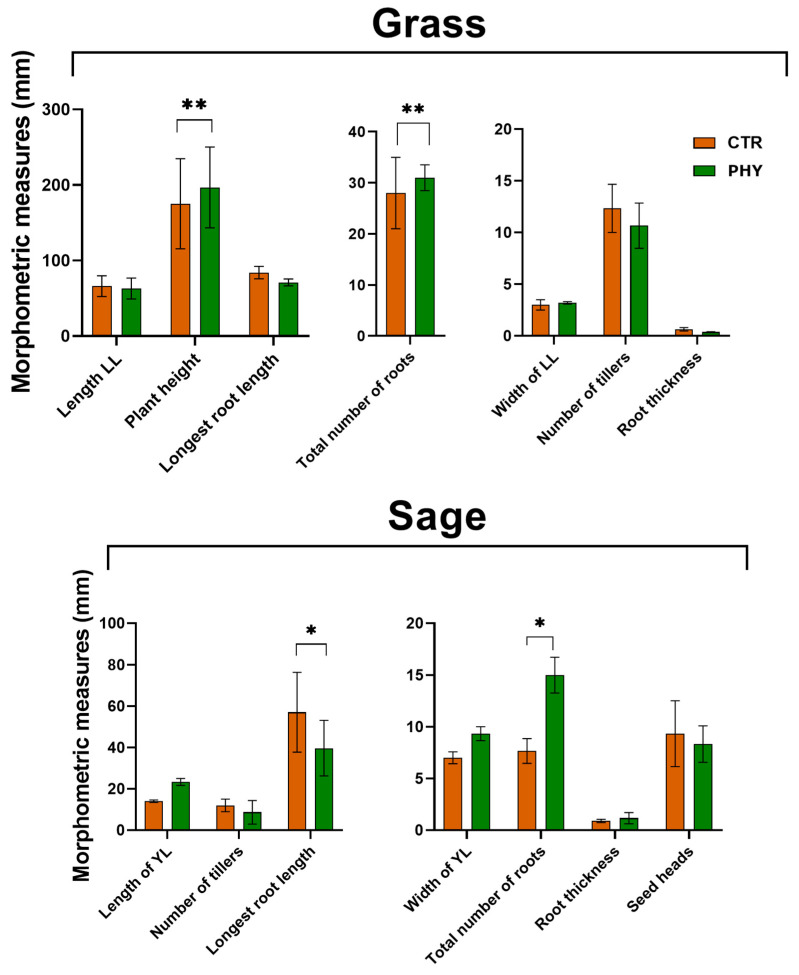
Plant morphometric measurements at week 20. The asterisk indicates significant change * = *p* < 0.05; ** = *p* < 0.01.

The morphometrics information from grass and the dominant weed (wild sage) was measured at week 20 (Figure 7) and after the summer wet season at 11 months (week 48) (Figures S4 and S5). At 20 weeks post-application, phytogenic liquid significantly increased grass height and the number of roots. In the sage plant, phytogenic liquid significantly increased the total number of roots and decreased the longest root length compared to the control, resulting in a shorter but more branched sage root system. Phytogenic liquid marginally decreased the number of branches and seed heads in sage and marginally increased the length and width of the youngest leaves.

Eleven months after the phytogenic liquid application, plant morphometrics were collected again. This time the changes in morphology were marginal. In phytogenic liquid plots, the grass and the grassroots were slightly shorter, but there were more tillers and roots, thicker than the roots of the grass growing in the control (Figure S5). Phytogenic liquid insignificantly suppressed dicot growth (Figure S4), including fewer roots and root thickness, contrary to week 20. Plants also had fewer branches and seed heads and shorter plant heights.

### 3.8. Dry Matter

**Figure 8:**
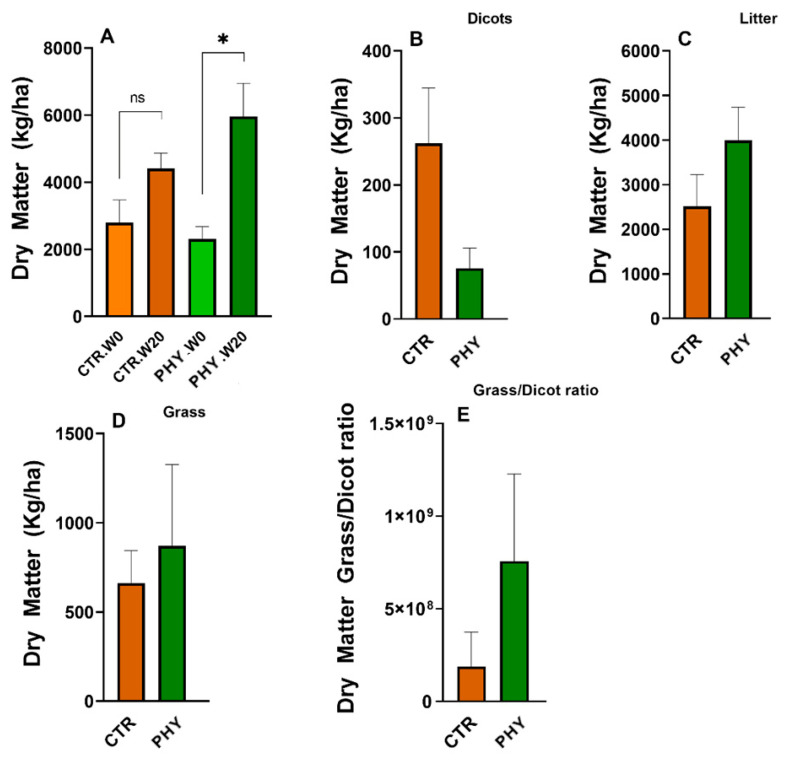
Dry matter differences between phytogenic liquid-treated and control plots. Panel (**A**) shows dry matter collected at week 0 and week 20. Panels (**B**–**D**) show dry matter collected 11 months after application. Panel (**E**) shows the grass/dicot ratio 11 months post-application. The asterisk in panel (**A**) indicates significant change (*p* < 0.05), ns = not significant.

Prior to PHY application at week zero, CTR and PHY plots had 2800 kg/ha and 2300 kg/ha of dry matter, respectively. This dry matter was then slashed so that the growth on all plots could restart evenly. Phytogenic liquid-treated plots significantly (*p =* 0.0116) harvested more biomass 20 weeks post-application compared to the dry matter prior to application (Figure 8A); this growth was not significant in CTR. After 20 weeks from the initial application, PHY plots had an average of 1567 kg/ha more dry matter than the CTR.

**Figure 9:**
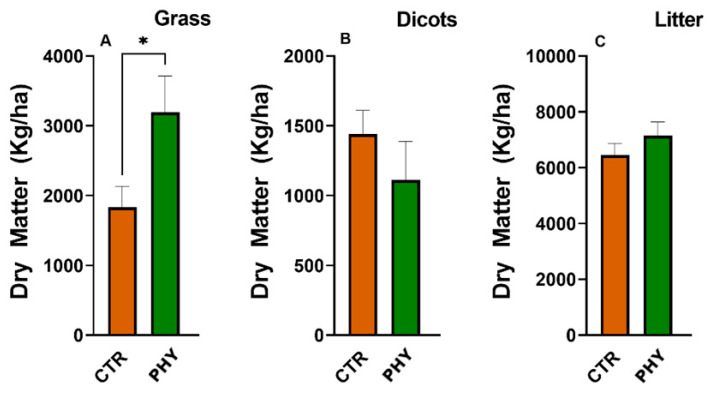
Dry matter differences between phytogenic liquid-treated and control plots 18 months post-application for grass (**A**), dicots (**B**) and litter (**C**). The asterisk indicates significant change (* = *p* < 0.05).

In preparation for the wet season, all plots were grazed to the ground and new pasture harvest was measured after the rains, 11 months after the initial application. Eleven months post-application, phytogenic liquid increased the average amount of grass by 209 kg/ha (Figure 8D), produced 1471 kg/ha more litter (Figure 8C), and significantly (*p* = 0.0495) inhibited dicotyledon growth with 186 kg/ha fewer weeds (Figure 8B) compared to the CTR. These changes in the pasture structure resulted in 1494 kg/ha more dry matter produced in phytogenic liquid plots in the second harvest. Moreover, the phytogenic liquid application improved the grass/dicot ratio to nearly three times higher than the control group after 11 months post-application (Figure 8E). The last sampling of dry matter was undertaken at 18 months post-application (Figure 9), and phytogenic liquid significantly affected grass (*p* = 0.0385) with 1360 kg/ha more biomass than in the control, which was even more significant and abundant than 11 months post-application. This result proved phytogenic liquid is effective for long-term pasture improvement, extending past one and a half years (Figure 9A). Meanwhile, it reduced dicots by 329 kg/ha, less than in the control, and increased litter by 693 kg/ha (Figure 9B,C). These changes resulted in 1724 kg/ha more biomass produced in phytogenic liquid-treated plots after 18 months of application.

Taken together, PHY-treated plots produced 1567, 1494, and 1724 kg/ha more total dry matter in the first, second, and third harvests, respectively, resulting in a total of 4785 kg/ha of the total dry matter gain compared with the untreated control. The cost of dry matter is complex to estimate as it depends on pasture quality and composition, but the local large hay bales containing 200–300 kg cost approximately AUD $50–$100 or moderately estimated as in the range of AUD $250–$500 per tonne, which estimates the cost of 4.8 tonnes of dry matter gain to be approximately in the range of AUD $1200 to $2400 per hectare.

The data from the second trial used to confirm the dry matter benefits (Figures S6 and S7) confirmed an increase in the grass of 1 t/ha, a reduction in dicots of 360 kg/ha, and 155 kg/ha more litter compared to the CTR, resulting in a total of 795 kg/ha more total dry biomass and an even more increased grass/weed ratio (Figures S6 and S7). In the first trial, the weeds were primarily herbaceous plants dominated by wild sage and basil. The weed structure in the second trial was more diverse and selective reduction in herbal (rather than brassica and legume-based weeds) was observed, but this was not further explored or quantified.

## 4. Discussion

Pasture dieback is a systematic ecological problem influenced by multiple factors, with no clear causes identified more than two decades after it first appeared. The short-term beneficial effects of some soil supplements, such as sea minerals [35] and a mix of humate and a common soil heavy metal remediation chemical [36], are promising, and their combinations with phytogenic liquid should be further investigated. We presented abundant evidence suggesting that blends of phytogens present a viable and highly promising option for the remediation and recovery of damaged and poor soils and plant promotion under stressful growing conditions.

Both CTR and PHY groups shared the same dominant phylum, *Actinobacteriota*, a group of gram-positive bacteria living in most of the common soil and freshwater. They are responsible for decomposing all forms of organic substances to facilitate the plant’s uptake of nutrients. *Actinobacteriota* members play a vital part in the rhizosphere microbial community in organic matter turnover and carbon cycle and are an essential part of humus formation [37]. Most of the other phyla in both groups are involved in C, N, and S cycles [38,39,40].

The microbial richness and diversity did not change significantly after phytogenic liquid application, but exciting temporal trends were observed, showing that both richness and diversity were restored faster in phytogenic liquid-treated plots in the weeks after the rain, reaching significantly higher diversity three weeks after the second rain event during week 10 after the application. This indicates that the phytogenic liquid was either still present in the soil or that it induced long-term beneficial changes in soil microbiota, which persisted through weeks of drought to provide better recovery after the rain. This dieback trial started at the peak of the dry season, and it would be interesting to compare these data with phytogenic liquid application during the wet season. Climate change is affecting Queensland [41]; the average temperature has increased by 1.5 °C compared to 1910 AD. However, farmers are more affected because the weather is becoming more unpredictable and extreme without affecting the average temperature [41], with more prolonged and severe droughts not reflected by the average rainfall. This study was undertaken in a dry sub-tropical climate with relatively high temperatures for most of the year and highly seasonal rainfall occurring primarily in the summer. It was reported that prolonged drought could irreparably damage the bacterial community, which is much more sensitive to heat than the fungal community [42], which disturbs the microbiome balance in the soil and prevents the recovery of bacterial networks. Our temporal alpha diversity data suggest that phytogenic liquid could assist this bacterial recovery under severe environmental conditions. 

The LEfSe analysis suggested biomarkers for both phytogenic liquid and the control group. Genera selected as representative of the control group are prevalent in extreme climate conditions, such as radiation, high temperature, and drought, and are indicators of poor soil conditions. *Rubrobacter* is a thermophilic radiation-resistant genus [43,44] and *Gaiella* is also found in extreme environments [45]. *Gaiellales* and *Rubrobacterales* are each represented by a single genus *Gaiella* and *Rubrobacter* and contain only a few species. They can adapt to survive in extreme environments, such as nutrient and energy-deprived deep South China Sea sediments. *Solirubrobacter* is a biomarker of poor and distressed soils [46]. Another genus reduced in phytogenic liquid plots and associated with control was *Acidothermus*. The most popular species of this genus is cellulolytic actinobacterial thermophile *Acidothermus cellulolyticus*, another indicator of temperature-stressed soils. Genome assembly for this species [47] investigated thermophilic genomic traits and hydrolytic enzymes capable of breaking down plant cell walls and degrading components in fungal cell walls [47]. Thermophilic bacteria and poor soil bacteria are the prevalent among the taxa reduced by phytogenic liquid application. This indicates the shift of microbiota from plant health-promoting beneficial rhizobial community members to those that can survive in extreme conditions and have minor, if any, benefits for plant growth, could play a role in pasture dieback pathology.

The known genera increased by phytogenic liquid include *Solirubrobacter*, *Bacillus*, *Planosporangium*, *Conexibacter*, *Bradyrhizobium*, *Geodermatophilus*, *Streptomyces*, and *Micromonospora* (Figure 6). Members of *Solirubrobacter* are beneficial to plant root endophytes [48], which could explain the morphometric benefits of phytogenic liquid, which significantly increased the total number of roots in both grass and sage (Figure 8) measured 20 weeks after application. *Bacillus* species are members of common soil bacterial communities, providing stress tolerance to plants as members of plant growth-promoting rhizobacteria. *Bacillus* spp. can be used on metal-contaminated soil as part of bioremediating procedures (reviewed in [49]). However, they may also have negative effects, such as suppressing other soil-beneficial microbes [49]. *Plantosporangium*, as the name suggests, are plant-associated rhizosphere bacteria [50] and *Conexibacter* are typical soil-dwelling bacteria [51] involved in nitrate reduction [52]. *Bradyrhizobium* are extremely well-researched, agriculturally significant soil rhizobial bacteria that can form symbiotic relationships with legumes and play a role in nitrogen fixation and detoxification. They are also an indicator of excellent soil health [53,54,55]. *Geodermatophilus* are common in grassland soil [56]. They are associated with the rhizosphere of the medicinal plant *Astragalus membranaceus* [57] and are able to survive in petroleum-polluted soil [58]. *Streptomyces* are highly abundant bacteria in typical healthy soil that produce two-thirds of clinically used antibiotics, such as neomycin, streptomycin and chloramphenicol, to name a few. *Streptomyces* are also a source of beneficial bioactive chemicals and enzymes and are considered the most biotechnology-beneficial soil bacteria. Their role in promoting plant growth and use as biofertiliser is reviewed by Olanrewaju et al. [59]. *Micromonospora* are recently discovered soil rhizosphere bacteria [60,61] highly relevant to forest and mangrove soils, and have been found in the grass (rice) rhizosphere [62].

To summarise, the taxonomic changes induced by phytogenic liquid include the reduction in genera implicated in poor and damaged soils, the significant promotion of plant- and soil-beneficial genera associated with the plant rhizosphere, and a range of other soil benefits. This is in agreement with measurable improvements in plant morphometrics and the amount of biomass. Our data also suggest that phytogenic liquid significantly alters several bacteria–mineral interactions. Altering any mineral–bacteria interactions would alter mineral–bacterial networks. Based on measured benefits for the plant, these network alterations could be critical and should be further investigated for the possible use of phytogenic liquid in the remediation of other contaminated or damaged soils. Considering that the phytogenic liquid is a blend of phytomolecules produced by plants to control soil and plant pathogenic microorganisms and insects, the ability of this product to selectively target plant pathogens and boost plant-beneficial microbiomes is not surprising, as it was the intended purpose of these plant-produced phytomolecules.

Pasture yield is a direct measurement of pasture health after any treatment. The phytogenic liquid treatment significantly harvested more biomass than the control, and these effects were very long-term, extending beyond 18 months post-application. The data suggest that grass promotion in terms of improved grass dry mass did not show any trends of reduction but instead became statistically significant 18 months after the application and after harvests via grazing and regrowth cycles. Phytogenic liquid treatment induced a shift in the grass–weed ratio, favouring grass production and produced more biomass under drought pressure. The predominant weeds in the paddocks used were herbs and a reduction in herbs was observed, but not in legumes and brassicas. This should be further investigated as it appears that high concentrations of phytogenic herbal bioactive compounds adversely affect other bioactive phytogen-producing herbal plants. If our observations are confirmed in controlled experiments, this could potentially be used in targeted weed control. This is significant because current weed control herbicide solutions are often toxic and environment-polluting, and usually eradicate all plants or all dicots indiscriminately. The ability of phytogenic liquid to selectively increase some and reduce other plants can be very useful in agriculture which is, now more than ever, looking for natural, non-toxic ways to tailor high-yielding plant agricultural systems. 

## 5. Conclusions

Our study provides the first evidence of the long-term recovery of pasture dieback-affected paddocks in Australia. The vast range of possible improvements phytomolecules can provide to pasture dieback, and other damaged, polluted, or poor-quality soils, should play a much more significant role in future sustainable agriculture where departure from chemical soil and plant treatments, supplements, pesticides, and fertilisers is identified as one of the major ecological goals. This resembles the deviation from antibiotics in livestock feed and the move toward natural phytogenic products. Rising temperature trends and climate change will challenge the soil microbial community and compromise its critical role in sustainable plant production. Our data show that phytogenic liquid can improve soil and plant health, overcoming shortfalls identified in phytogenic products, mainly volatility and fast evaporation and the need for frequent re-application. In livestock, phytogenic supplements are provided preventatively, and their benefits are most visible during highly stressful events, such as disease outbreaks or heat stress. We suggest further research should focus on the ability of stable phytogen products in agronomy production systems to better cope with climate change and other environmental stresses.

## Figures and Tables

**Table 1:** Multivariate analysis of beta diversity.

Distance	Significance of	*p*-Value	Significance
Weighted UniFrac	Treatment	0.247	ns
	Time	0.001	***
	Plot	0.009	**
Unweighted UniFrac	Treatment	0.241	ns
	Time	0.001	***
	Plot	0.012	*
Phylum level Bray–Curtis	Treatment	0.0273	*
	Time	0.002	**
	Plot	0.466	ns
Genus level Bray–Curtis	Treatment	0.384	ns
	Time	0.001	***
	Plot	0.001	***

There was a significant (*p* = 0.049) interaction between the treatment and time at the genus level, * = *p* < 0.05; ** = *p* < 0.01; *** = *p* < 0.001; ns = not significant.

**Table 2:** Phyla and known genera with the highest contribution to CTR–PHY group dissimilarity.

Phylum	Contribution (%)
*Firmicutes*	19.38
*Proteobacteria*	10.14
*Actinobacteriota*	9.59
*Verrucomicrobiota*	9.32
*Acidobacteriota*	7.53
*Myxococcota*	6.44
*Chloroflexi*	6.42
*Gemmatimonadota*	5.73
Genus	**Contribution (%)**
*Bacillus*	7.19
*Rubrobacter*	4.96
*Solirubrobacterales 67-14*	3.06
*Solirubrobacter*	2.74
*Acidothermus*	2.39
*Conexibacter*	2.31
*Gaiella*	2.28
*MB-A2-108*	2.26
*Micromonospora*	2.21
*Planosporangium*	2.11
*Candidatus Udaeobacter*	1.89
*Geodermatophilus*	1.77
*Jatrophihabitans*	1.45
*Pseudonocardia*	1.25
*Chloroflexi TK10*	1.22
*Chloroflexi JG30-KF-CM45*	1.21
*Streptomyces*	1.07
*Pyrinomonadaceae RB41*	1
*Rhodoplanes*	0.93
*IMCC26256*	0.9
*Actinoallomurus*	0.81
*Actinoplanes*	0.81

## Data Availability

Data associated with this study are deposited in the NCBI database under the accession number PRJNA887675 (https://www.ncbi.nlm.nih.gov/bioproject/?term=PRJNA887675 (accessed on 1 December 2022)).

## Supplementary Materials

The following supporting information can be downloaded at: https://www.mdpi.com/article/10.3390/microorganisms11030561/s1, Figure S1: Temporal distribution of phyla; Figure S2: Relative abundance at phylum level; Figure S3: Relative abundance of biomarkers; Figure S4: Morphometric measurements in dicots six months after treatment; Figure S5: Morphometric measurements in monocot six months after treatment; Figure S6: Dry matter differences between healthy, phytogenic liquid-treated, and control plots. Data from the second trial; Figure S7: Grass/dicots ratio among healthy, phytogenic liquid-treated, and pasture dieback plots.
