# Supplementary material for: Application of Phytogenic Liquid Supplementation in Soil Microbiome Restoration in Queensland Pasture Dieback
Source: Microorganisms. 2023 Feb 23;11(3):561. doi: 10.3390/microorganisms11030561 (PMC10054416; doi:10.3390/microorganisms11030561)
Supplement: Supplementary file 1 [file microorganisms-11-00561-s001.zip › microorganisms-2240475-supplementary.pdf]

microorganisms

Supplementary Material

Application of phytogenic liquid supplementation on soil microbiome restoration in Queensland pasture dieback

Supplementary Material

Xipeng Ren[1], Maria M. Whitton[1], Sung J. Yu[1], Tieneke Trotter[1], Yadav S. Bajagai[1] and Dragana Stanley[1,*]

microorganisms

MDPI

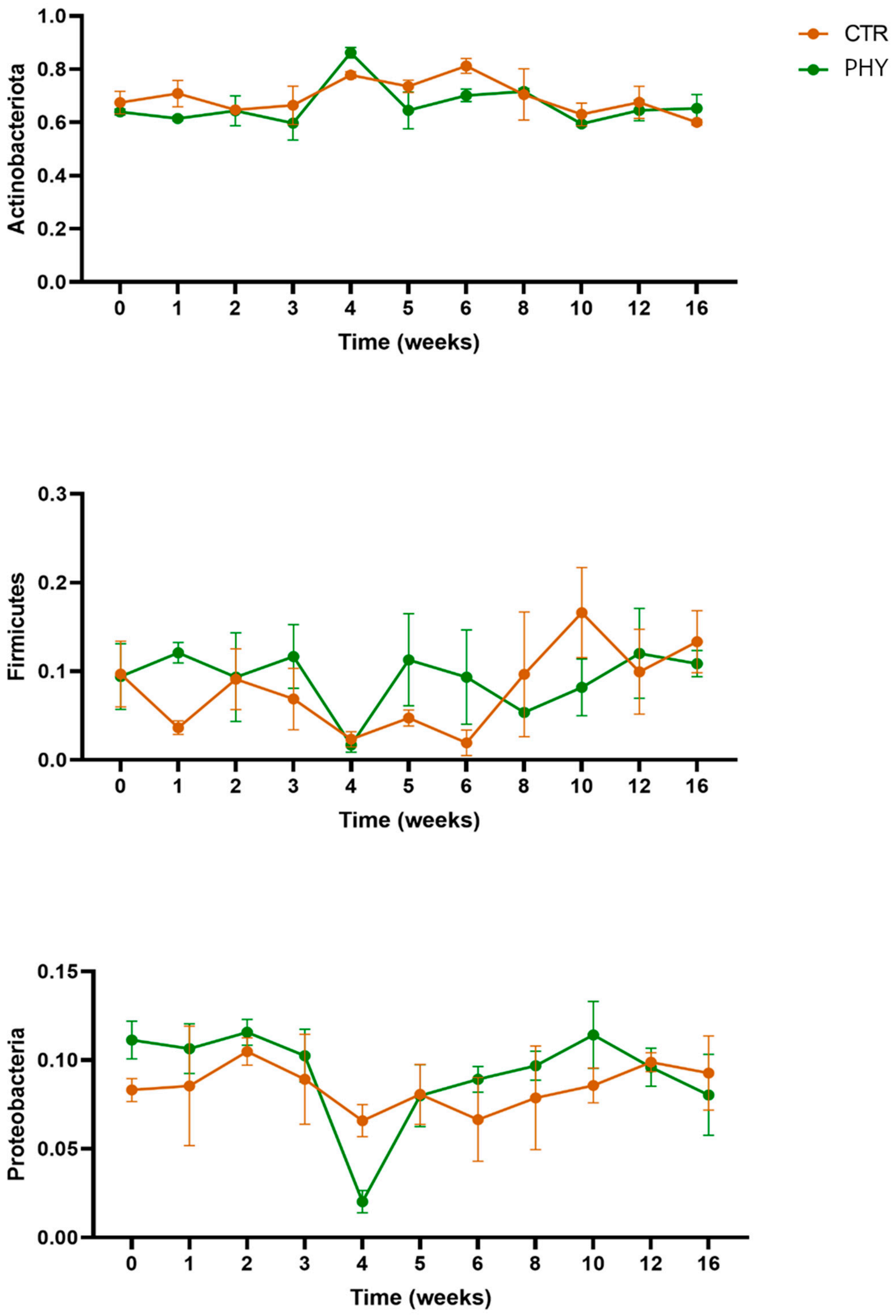

Figure S1. Temporal distribution of phyla

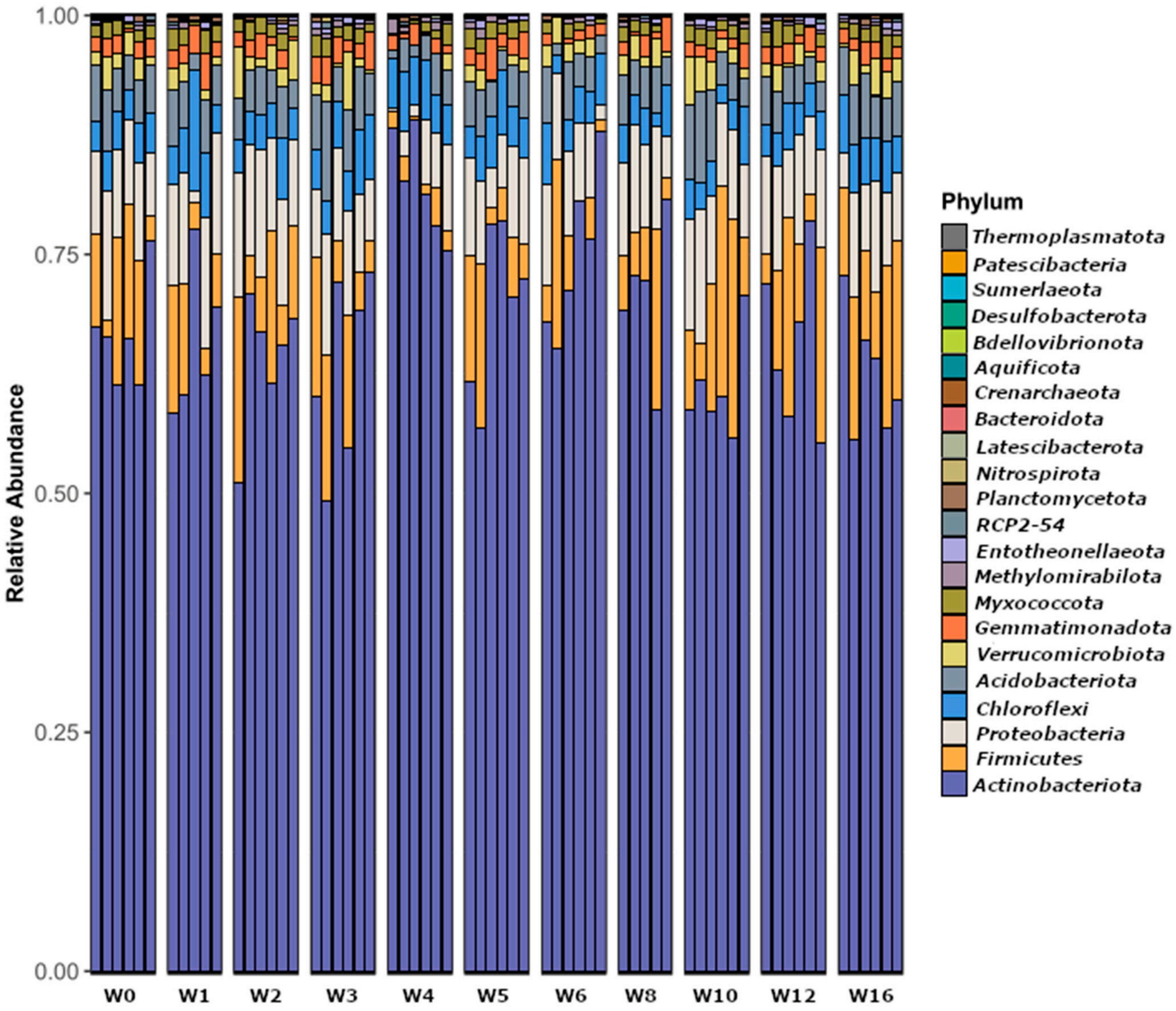

Figure S2. Relative abundance at phylum level

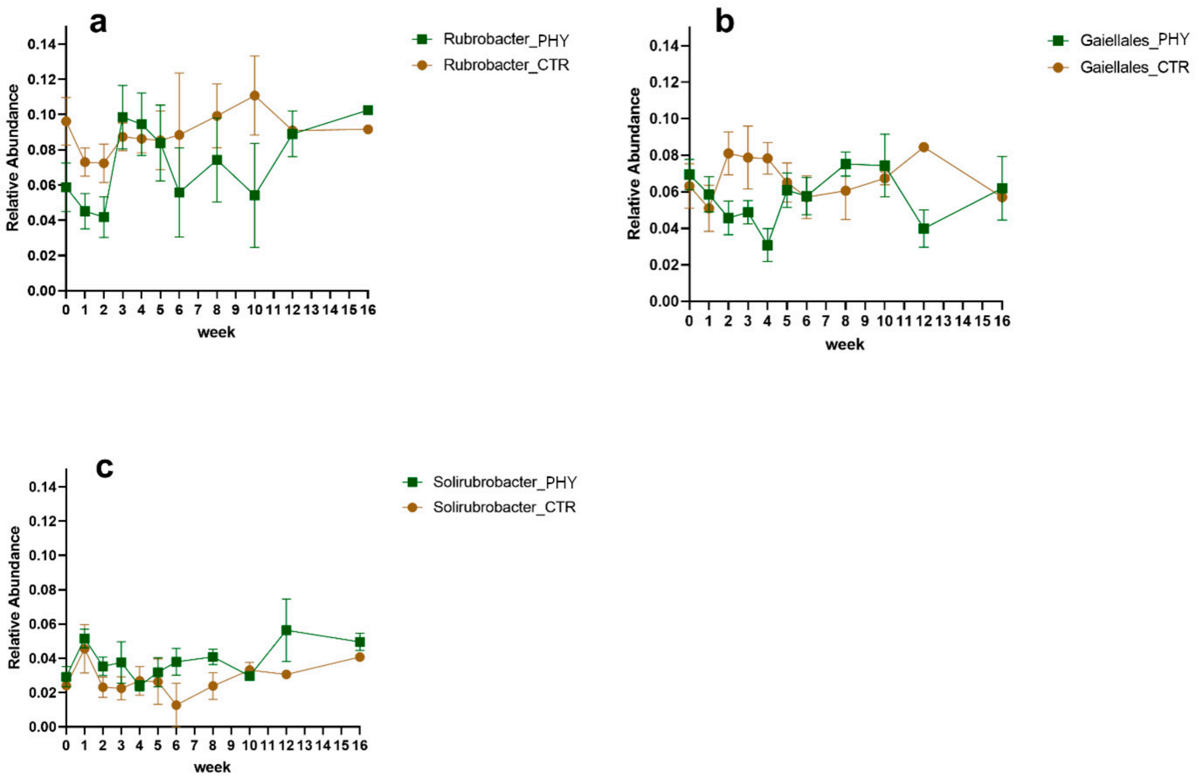

Figure S3. Relative abundance of biomarkers.

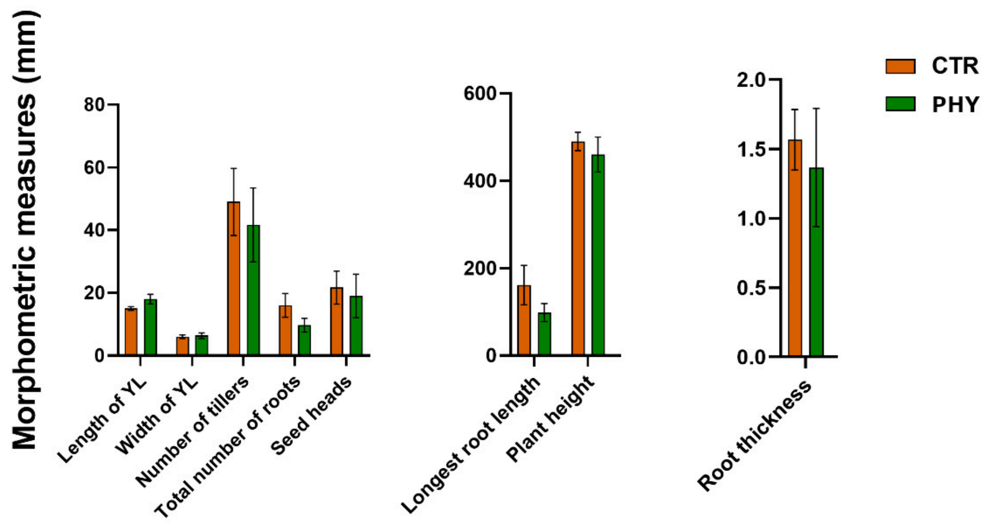

Figure S4. Morphometric measurements in dicots six months after treatment.

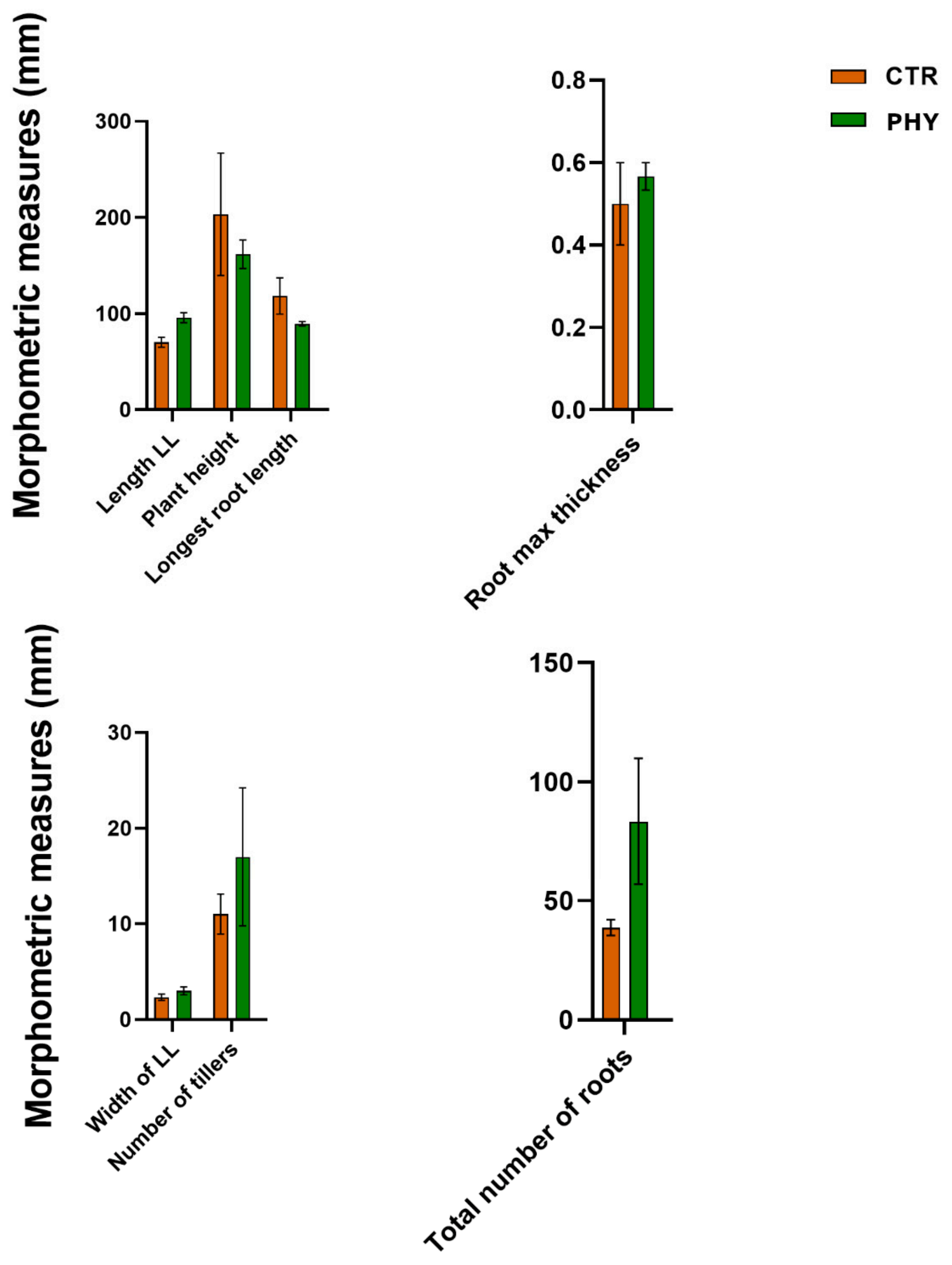

Figure S5. Morphometric measurements in monocot six months after treatment.

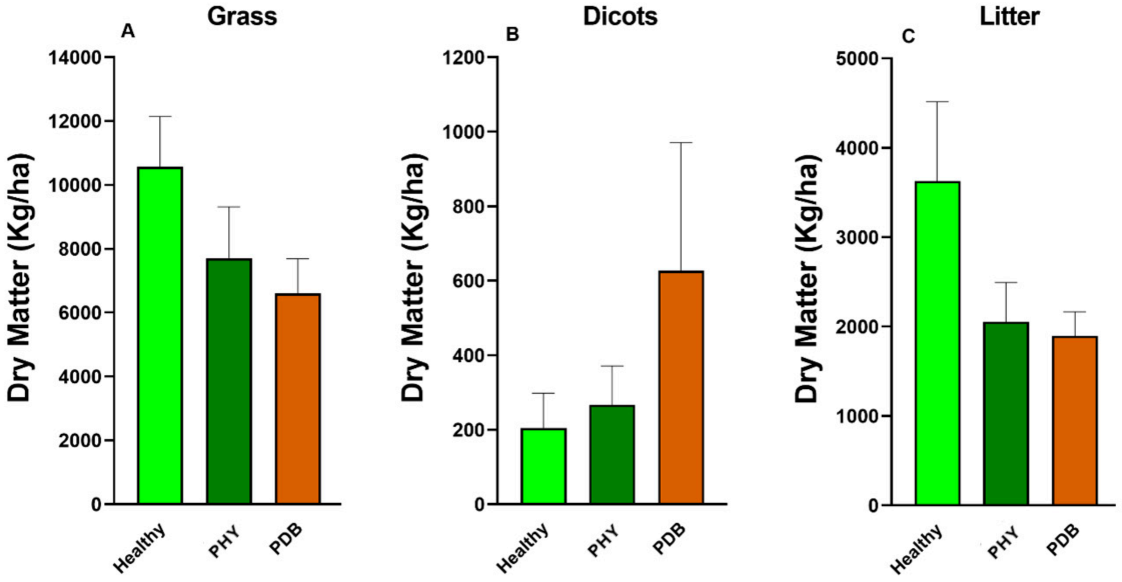

Figure S6. Dry matter differences between healthy, phytogenic liquid treated and control plots. Data from the second trial.

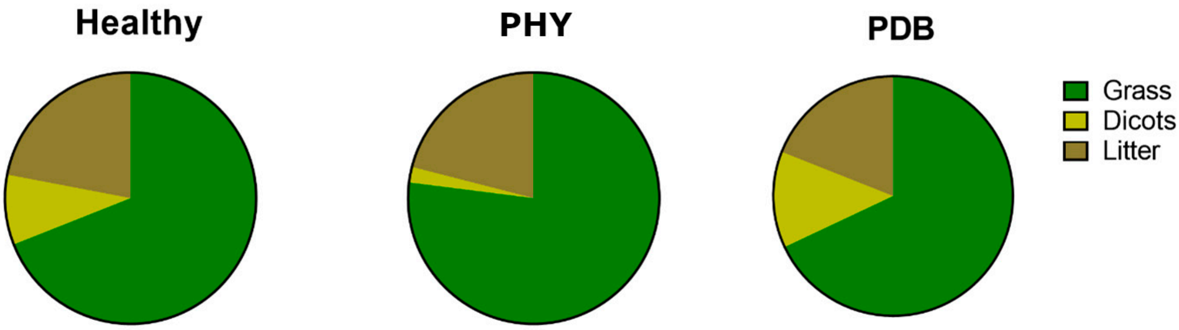

Figure S7. Grass/dicots ratio among healthy, phytogenic liquid treated and pasture dieback plots. Data from the second trial.
